# Measuring the Performance of Attention Networks with the Dalhousie Computerized Attention Battery (DalCAB): Methodology and Reliability in Healthy Adults

**DOI:** 10.3389/fpsyg.2016.00823

**Published:** 2016-06-07

**Authors:** Stephanie A. H. Jones, Beverly C. Butler, Franziska Kintzel, Anne Johnson, Raymond M. Klein, Gail A. Eskes

**Affiliations:** ^1^Cognitive Health and Recovery Research Laboratory, Department of Psychiatry, Dalhousie UniversityHalifax, NS, Canada; ^2^Research Services, Nova Scotia Health AuthorityHalifax, NS, Canada; ^3^Cognitive Science Laboratory, Department of Psychology and Neuroscience, Dalhousie UniversityHalifax, NS, Canada; ^4^Affiliated Scientist, Medical Staff, Nova Scotia Health AuthorityHalifax, NS, Canada

**Keywords:** computerized assessment, attention, orienting, alerting, executive function

## Abstract

Attention is an important, multifaceted cognitive domain that has been linked to three distinct, yet interacting, networks: alerting, orienting, and executive control. The measurement of attention and deficits of attention within these networks is critical to the assessment of many neurological and psychiatric conditions in both research and clinical settings. The Dalhousie Computerized Attention Battery (DalCAB) was created to assess attentional functions related to the three attention networks using a range of tasks including: simple reaction time, go/no-go, choice reaction time, dual task, flanker, item and location working memory, and visual search. The current study provides preliminary normative data, test-retest reliability (intraclass correlations) and practice effects in DalCAB performance 24-h after baseline for healthy young adults (*n* = 96, 18–31 years). Performance on the DalCAB tasks demonstrated *Good* to *Very Good* test-retest reliability for mean reaction time, while accuracy and difference measures (e.g., switch costs, interference effects, and working memory load effects) were most reliable for tasks that require more extensive cognitive processing (e.g., choice reaction time, flanker, dual task, and conjunction search). Practice effects were common and pronounced at the 24-h interval. In addition, performance related to specific within-task parameters of the DalCAB sub-tests provides preliminary support for future formal assessment of the convergent validity of our interpretation of the DalCAB as a potential clinical and research assessment tool for measuring aspects of attention related to the alerting, orienting, and executive control networks.

## Introduction

Attention is a multifaceted cognitive domain that is required for efficient perception, learning, memory, and reasoning. Different aspects of attention have been conceptualized in a number of models, such as Theory of Visual Attention (TVA, a computational model of selective attention, Bundensen, 1990), Working Memory (Baddeley and Hitch, 1974, updated in Baddeley, 2012), Cowan's information processing model (Cowan, 1988, 1998) and the attentional trace model of auditory selective attention (Naatanen, 1982). These, and other aspects of attention were integrated into a neurocognitive framework of an attention system that involves separate but interacting attentional brain networks underlying the attentional functions of alerting, orienting (selection), and executive control (e.g., Posner and Petersen, 1990; Fan et al., 2002; Petersen and Posner, 2012). *Alerting* or vigilance refers to the ability to develop and sustain a state of mental readiness, which consequently produces more rapid selection, detection, and responses to relevant stimuli in the environment (Posner and Petersen, 1990). The alerting system has been measured with continuous performance and vigilance tasks in which the participant is asked to continuously respond to sequentially presented stimuli with or without warning signals prior to the target (phasic alertness vs. tonic alertness); these tasks are thought to activate right hemisphere frontal and dorsal parietal regions related to the neuromodulator norepinephrine (c.f. Sturm and Willmes, 2001; Petersen and Posner, 2012 for a full review). The *orienting* of attention involves the selection of a stimulus or spatial location in the environment in order to process that information more fully. Orienting attention requires that attention be disengaged from its current focus, moved, and then re-engaged on the selected location/stimulus. While Posner's original orienting system was associated with cholinergic systems involving the frontal lobe, parietal lobe, superior colliculus, and thalamus (Posner et al., 1982; Posner and Petersen, 1990), recent evidence argues for two separate but interacting orienting networks: (1) a bilateral dorsal system including the frontal eye fields and intraparietal sulci related to rapid strategic control over attention and; (2) a strongly right-lateralized ventral system including the temporoparietal junction and ventral frontal cortex related to breaking the focus of attention and allow switching of attention to a new target (Corbetta and Shulman, 2002; Petersen and Posner, 2012). Spatially-cued target detection tasks and visual search tasks are typically used to assess attentional orienting. Finally, in terms of *executive control* function, evidence indicates the presence of at least two relatively independent executive networks: (1) a fronto-parietal network, distinct from the orienting network, that is thought to relate to task initiation, switching, and trial-by-trial adjustments, and; (2) a midline cingulo-opercular and anterior insular network related to maintenance across trials and maintenance of task performance as a whole, i.e., set maintenance, conflict monitoring, and error feedback (Petersen and Posner, 2012, see Figure 2B; Dosenbach et al., 2007, see Figure 4).

Given the above framework and the overarching effect that attentional impairment can have on the successful completion of day to day activities and overall quality of life (e.g., Bronnick et al., 2006; Barker-Collo et al., 2010; Cumming et al., 2014; Middleton et al., 2014; Torgalsbøen et al., 2015), valid and reliable measurement of attention is important for addressing attention impairment in both research and clinical care of psychiatric and neurologic patients. While most standardized “paper-and-pencil” measures used in the neuropsychological assessment of attention allow comparison of an individual's performance to a healthy normative sample (as well as patient samples), it can be difficult to isolate specific domains of attention as conceptualized in Posner's model *per se* (e.g., Chan et al., 2008). Therefore, a supplementary or alternative procedure to standardized neuropsychological testing is necessary to provide reliable and sensitive assessment of attentional function. Computer-based cognitive testing procedures have been developed as one option.

The Attention Network Test (ANT; Fan et al., 2002) is one such computerized measure of attention designed to quantify the efficiency of the vigilance, orienting and executive control networks through the combination of a cued reaction time task (Posner, 1980) and a flanker task. Participants are instructed to respond to the direction of an arrow stimulus flanked by congruent, incongruent, or neutral stimuli, following the presentation of one of four cue conditions. Difference values between the various cue and flanker conditions can be used as a quantitative measure of the efficiency of the vigilance, orienting, and executive control attention networks (cf Fan et al., 2002 for a full review). Subsequent versions of the ANT also allow an assessment of the interactions among the attention networks (ANT-Interactions, Callejas et al., 2005; ANT-Revised, Fan et al., 2009). The ANT and ANT-I have been used in a variety of populations, including children, healthy adults of varying ages, and a variety of clinical populations and allow for comparisons across attentional networks due to their integrated nature and brevity (Fernandez-Duque and Black, 2006; Adolfsdottir et al., 2008; AhnAllen et al., 2008; Ishigami and Klein, 2010; Ishigami et al., 2016; see review in MacLeod et al., 2010). While use of the ANTs has proved valuable for exploration of attention mechanisms in normal and clinical populations, there are limitations to these tests. In order to limit test time, the assessment of alerting, orienting, and executive control is dependent upon a single difference measure producing a network score, with alerting and orienting network RT scores showing lower than ideal reliability across studies (ranging from 0.20 to 0.61) and the executive network RT score showing better reliability (ranging from 0.65 to 0.81; Fan et al., 2002; MacLeod et al., 2010). In addition, investigations of the validity of the ANT are limited, but Ishigami et al. (2016) found that while the executive network score was a significant predictor of conflict resolution, and verbal memory retrieval, no associations were found between the alerting and orienting network scores and other standardized tests of attention. Likewise, comparisons between the ANT and an assessment based on TVA also yielded no significant correlations (Habekost et al., 2014). Whether this lack of correlation attests to the unique measurement properties of the ANT, or its lack of validity is unknown at this point.

Thus, to extend this tri-partite neurocognitive approach and to provide multiple yet integrated measures that can be compared across networks, we developed the Dalhousie Computerized Attention Battery (DalCAB; Butler et al., 2010; Eskes et al., 2013; Rubinfeld et al., 2014; Jones et al., 2015). The DalCAB is a battery of eight computerized reaction time tests, each test previously used individually in cognitive neuroscience research to measure multiple attentional functions within the vigilance, orienting and executive control attention networks. These tasks reflect concepts of attentional functions frequently studied in normal populations as well as in those affected by psychiatric or neurological disease/injury but, to date, have never been combined and integrated using standardized stimuli^1^ in a single, computerized battery.

The DalCAB includes the following tasks: simple and choice reaction time tasks to measure vigilance, a visual search task to measure orienting, and go/no-go, dual task, flanker, item working memory, and location working memory^2^ tasks to target executive control functions. The DalCAB emphasizes reaction time and accuracy measures that have been consistently related to attentional functions and can be sensitively and robustly measured (e.g., Sternberg, 1966; Wicklegren, 1977; see below). A description of all DalCAB tasks, the effects of interest within each task and literature relevant to each task are presented in Table 1. We have omitted the description of the location working memory task in the section below ^2^.

**Table 1:** **DalCAB task descriptions, outcome measures, and task-related variables and effects of interest**.

**Task and description**	**Outcome measures**	**Task variable**	**Effect of interest**	**Effect obtained**
**SRT:** Respond to each stimulus, with varying response-stimulus intervals.	RT^b^ % correct	Response-stimulus interval (three, between 500 and 1500 ms).	*Temporal preparation effect*: Faster RTs with longer RSIs (e.g., Henderson and Dittrich, 1998; Vallesi et al., 2013 for review).	Yes
**GNG:** Respond to single target color, with high (80%) or low (20%) frequency targets.	RT^b^ % correct %FA	Proportion Go Trials (80, 20%).	*Response inhibition*: Faster RTs and more false alarms (button press on a no-go trial) when go frequency is high (80%). *Sustained attention*: More omissions when go response is low (20%; e.g., Carter et al., 2013).	Yes
**CRT:** Indicate the color of each stimulus (2-choice responses; 50% each choice).	RT^b^ % correct	Trial type (switch, no-switch in response).	*Switch effect*: Slower RTs on trials that require a change in response from the previous trial (e.g., Bailon et al., 2010).	Yes
**Dual Task:** Complete CRT while silently counting the number of each color of stimuli presented. Count probe for one color at the end of each set.	RT^b^(CRT) % correct CRT % correct counting	CRT trial type (switch, no-switch); Counting set size (8, 12).	*Switch effect in the CRT task*: Slower RTs on trials that require a change in response from the previous trial.	Yes
			*Working memory load effect*: Slower choice RTs overall when compared to the single task CRT (Baddeley and Della Sala, 1996; Hommel and Doeller, 2005).	Yes
**Flanker:** Indicate shape of a central target flanked above and below by same- or different-shaped distractors (50% congruent with flanker shapes).	RT^b^ % correct	Flanker Congruency (Congruent, Incongruent).	*Congruency effect:* Slower RTs and more errors when flanking stimuli do not match the target (incongruent trials; Eriksen and Eriksen, 1974; Ishigami and Klein, 2010, 2011).	Yes
**IM:** Indicate whether a probe item was present or absent in a preceding study set of 2–6 items (50% present).	RT^b^ % correct	Set size (three, between 2 and 6); Trial type (present, absent).	*Set size effect*: Slower RTs for larger set sizes (Sternberg, 1969; Poewe et al., 1991; Ferraro and Balota, 1999).	Yes
**LM:** Indicate whether a probe location was present or absent in preceding study sets of 2–6 spatial locations (50% present).	RT^b^ % correct	Set size (three, between 2 and 6); Trial type (present, absent).	*Set size effect*: Slower RTs for larger set sizes (Sternberg, 1969; Poewe et al., 1991; Ferraro and Balota, 1999);^1^	N/A^2^
**VS:** Locate and indicate orientation (upright vs. inverted; 50% each) of a target among different shape distractors that are a different color (feature search) or the same color (conjunction search) as the target.	RT^b^ % correct	Set size (three, between 6 and 18); Search type (feature, conjunction).	*Set size effect*: Slower RTs for larger set sizes in conjunction search (Treisman and Gelade, 1980; Davis and Palmer, 2004).	Yes

b *Reaction time derived from correct trials only*.

### Simple reaction time (SRT, vigilance)

SRT is used to measure response readiness and motor reaction time (RT) to the onset of all stimuli presented. Previous research has indicated that the SRT task involves attention-demanding pre-trial vigilance (for stimulus onset and/or response initiation), and performance is affected by transient warning signals and tonic arousal changes (Petersen and Posner, 2012; Steinborn and Langner, 2012). In addition, if the interval between stimuli is varied, faster RTs are observed with longer response-stimulus intervals (RSIs), a phenomenon called the temporal preparation effect or fore-period effect (Vallesi et al., 2013). SRT is slowed in normal aging and in patients with frontal lobe alertness deficits (Godefroy et al., 2002, 2010). When the RSI is variable, older participants have shown a reversal in the fore-period effect related to decreased right prefrontal activation (Vallesi et al., 2009). SRT is also differentially slowed by dividing attention and by neurological disorders such as Parkinson's disease that affect frontal systems function (c.f. Henderson and Dittrich, 1998 for a full review).

### GO/NO-GO (GNG, executive control)

Frequently used to measure response inhibition or sustained attention, the GNG task employs a continuous stream of two different stimuli for which a binary decision must be made, such that one stimulus type requires a response (go) and the other stimulus type requires the participant to withhold a response (no-go). Response inhibition performance is measured by the percent of responses on no-go trials (false alarms; commission errors), particularly when go trials are more frequent than no-go trials (Carter et al., 2013). In contrast, sustained attention is measured by response performance on go trials (omission errors and reaction time), particularly when go trials are less frequent compared to no-go trials (also referred to as a vigilance or traditionally formatted task or TFT; Carter et al., 2013). Response inhibition deficits are seen in acquired brain injury, bipolar disorder, Parkinson's disease, and other neurological disorders (e.g., Claros-Salinas et al., 2010; Dimoska-Di Marco et al., 2011; Fleck et al., 2011). While sustained attention or vigilance decrements have frequently been studied in sleep disordered breathing (Kim et al., 2007), they have also been shown in Parkinson's disease (Hart et al., 1998).

### 2-Choice reaction time (CRT, vigilance)

Often used to measure decision time and response selection, the CRT task requires different responses for each of two different stimuli presented in a continuous stream (e.g., left button for red stimuli, right button for black stimuli). Errors, reaction time decrements over time and switch costs, calculated as the difference in reaction time between trials that require a switch in response category (switch trials) vs. non-switch trials, are often used to determine deficits in decision and response selection time. CRT responses are slowed in dementia, stroke, multiple sclerosis, and other neurological disorders (e.g., Bailon et al., 2010; Stoquart-Elsankari et al., 2010).

### Dual task (executive control)

Often used to measure attentional control, attentional load effects, and interference, dual-task paradigms require the participant to perform two tasks simultaneously. By comparing the dual task performance to single task performance the degree of dysfunction related to attentional load or interference by the secondary task (i.e., dual task cost) can be measured (Baddeley and Della Sala, 1996). In dual task studies, addition of a concurrent secondary task greatly reduces primary task performance in many neurological disorders, including Alzheimer's disease, traumatic brain injury, and Parkinson's disease (Dalrymple-Alford et al., 1994; Della Sala et al., 1995, 2010).

### Flanker (executive control)

The flanker task is used as a measure of selective attention, filtering, and/or conflict resolution and performance is considered to reflect the executive attention network. In this task a central target stimulus is presented with flanking stimuli (flankers) on two sides that are either the same as (congruent) or different than (incongruent) the central target stimulus. The participant must make a decision and response regarding a feature of the central stimulus (e.g., red or black) while ignoring/filtering the flanking stimuli. In healthy adults, reaction times are slowed and accuracy is lower on trials in which the flankers are incongruent with the target compared to when the flankers are congruent with the target (i.e., the reaction time interference effect), although the effect diminishes with practice (Ishigami and Klein, 2010, 2011). The RT interference effect has also been noted to increase with increasing age (Salthouse, 2010), although accuracy effects in older adults have been shown to be smaller than those of younger adults (D'Aloisio and Klein, 1990), suggesting that the larger RT interference effects in older adults are related to a response bias favoring accuracy over speed on incongruent trials. In patient groups, larger RT interference effects (i.e., impaired conflict resolution) have been associated with elevated symptom severity in adults with post-traumatic stress disorder (Leskin and White, 2007) and borderline personality disorder (Posner et al., 2002). In addition, while interference effects are significantly large in dementia patients (Fernandez-Duque and Black, 2006; Krueger et al., 2009), across many neurodegenerative diseases, accuracy and reaction time performances on the flanker task are associated with different patterns of regional brain atrophy (Luks et al., 2010).

### Item working memory (executive control)

Used to measure working memory capacity and scanning efficiency, the item working memory task presents a set of stimuli to be remembered. This stimulus set is followed after a delay by a probe stimulus. The participants' task is to indicate whether the probe stimulus was present in the previously viewed set. In healthy individuals, as the number of items in the set increases, decision accuracy decreases, and the time required to make a determination about the probe stimulus increases (Sternberg, 1969). In normal aging, memory scanning slows as evidenced by increases in slope and intercept on this task (i.e., the increase in reaction time for each additional item in the set, representing memory scanning rate, and the point at which the set size regression line crosses the y-axis, representing the speed of combined encoding, decision making and response selection aspects of the task), and these measures increase further in individuals with dementia (Ferraro and Balota, 1999). Compared to healthy controls, patterns of memory scanning speed and decision accuracy differ depending on the neurological disorder and the medication state studied. For example, patients with multiple sclerosis are equally accurate but have slower memory scanning speed (Janculjak et al., 1999), while Parkinson's patients are less accurate and have “normal” memory scanning speed unless they are on medication (levodopa; Poewe et al., 1991). A description of the location working memory task can be found in Table 1, but will not be discussed here.

### Visual search (orienting and selection)

The visual search task has been used to measure spatial orienting and selection. In this task, a target is presented within distractor sets of various sizes and the participant's task is to respond to the target (either a detection or identification response; Davis and Palmer, 2004). When the target stimulus is very different from the distractors (e.g., a different color) response to the target is fast and independent of the number of distractors (feature search). In contrast, if the target and distractors share some (but not all) features in common with the target, search is slower and influenced by distractor set size (conjunction search; Treisman and Gelade, 1980). Compared to healthy controls, a differential effect of performance has been found across feature and conjunction search types depending on the neurological disorder. For example, overall performance on both search types has been shown to decrease (i.e., increased RTs and error rates) in persons with mild cognitive impairment (e.g., Tales et al., 2005) and Alzheimer's Disease (e.g., Foster et al., 1999; Tales et al., 2005). In contrast, while persons with Schizophrenia have been shown to exhibit slowed RTs in conjunction search, little difference is found between patients and controls for feature search. Computerized visual search paradigms have also been shown to differentiate between stroke patients and healthy controls and between stroke patients with and without spatial neglect.

The purpose of this study is twofold. First, we report normative data for seven of the DalCAB tasks^2^ obtained from a preliminary sample of healthy adults (*n* = 100, 18–31 years of age), including analyses of individual tasks to determine the presence or absence of the expected pattern of effects within each task. These data are intended to serve as pilot evidence for further exploration of the convergent validity of our interpretation of the DalCAB^3^, Second, we report the test-retest reliability and practice effects from our healthy adult sample (*n* = 96).

## Methods

### Participants

One-hundred and three healthy adults were enrolled in the study. Demographic information for participants included in the analyses is presented in Table 2. Participants were recruited through an undergraduate research participant pool at Dalhousie University in exchange for partial course credit or through the use of flyers and notices posted in and around the Dalhousie University community (e.g., library, coffee shops, etc.) in exchange for a per-session dollar amount. All participants provided informed consent following procedures approved by the Capital District Health Authority Research Ethics Board in Halifax, Nova Scotia, Canada. In advance of participation, all participants were screened through self-report for past or current neurological disorders, loss of consciousness for more than 5 min, history of neuropsychiatric disorders and current use of antidepressant or anti-anxiolytic medications known to influence cognitive performance. Two participants were excluded in advance of participation due to their medication use and data from one participant were removed from analysis due to the use of an incorrect testing procedure. Four participants also withdrew or were removed after the baseline session due to noncompliance with the study protocol. Thus, 100 participants completed the DalCAB at baseline and 96 completed the DalCAB at 24 h after baseline (see Table 2 for sample demographic information).

**Table 2:** **Participant demographic information for healthy adults completing the DalCAB at the baseline and 24-h testing sessions**.

	**Baseline**	**24-h**
N	100	96
Mean age (SD)	21.8 (3.1) years	21.7 (3.1) years
Number female (%)	64 (64%)	60 (62.5%)
Number right-handed (%)	92 (92%)	87 (90.6%)
Mean education (SD)	14.8 (2.4) years	14.8 (2.4) years

### Apparatus and procedure

**Figure 1:**
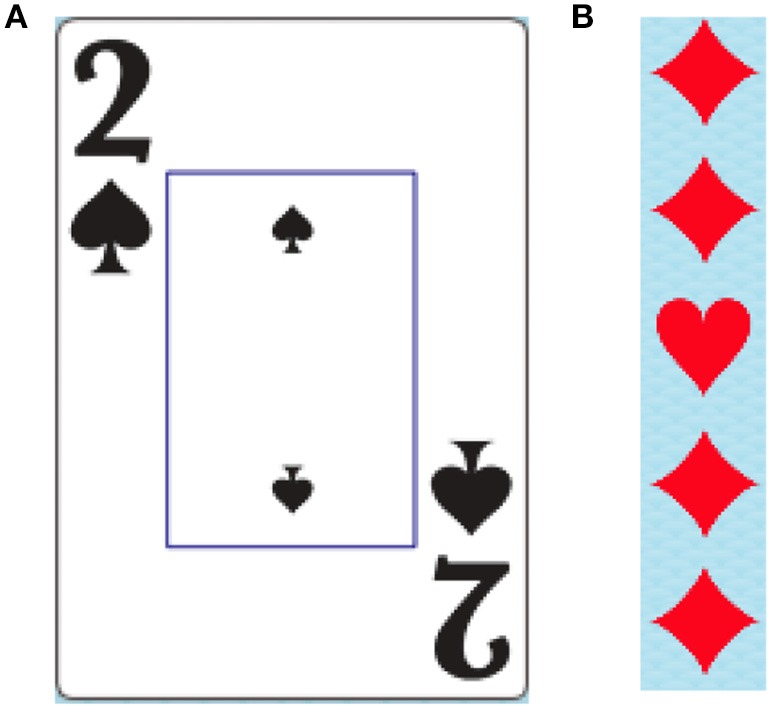
**Example of card suit stimuli used in the DalCAB tasks**. **(A)** Card as shown in simple response, inhibition, decision speed, dual task and item working memory tasks. **(B)** Card suit shapes as shown in the flanker tasks. Card suit shapes like those in **(B)** are also used in the visual search task.

All tasks contained within the DalCAB employ a variation of playing cards or card suit stimuli (example stimuli shown in Figure 1). Stimuli were presented on Apple Computers (iMac G3 and iMac with a 27-inch monitor). Participants' responses to stimuli were collected using a two-button mouse. Participants were seated 50 cm away from the computer monitor on which all instructions, practice trials, and experimental trials for the DalCAB tasks were presented. For each task, the experimenter read the instructions printed on the screen to the participant. Speed and accuracy were equally emphasized in all task instructions. Participants were then given the opportunity to practice the task (12–36 trials, depending on the task) during which time they received auditory and/or visual feedback about their performance. Once comfortable with the nature of the task, the participant completed the experimental trials without auditory or visual feedback about their performance. All participants completed the DalCAB tasks in the same order (as described above). Programming changes during the development of the Location working memory task resulted in a small sample size (*n* = 15); thus, the Location working memory task will not be presented in this report^2^. A description of all tasks included in the DalCAB are presented in Table 1 (see also Jones et al., 2015). Each DalCAB session, including practice and experimental trials, took ~1-h to complete.

## Data analysis

Mean reaction time (RT) in milliseconds and accuracy measures (% correct, % false alarms) were collected. Reaction times less than 100 ms were coded as anticipatory and were excluded from analysis. Reaction times greater than the maximum reaction time (varied by task) were coded as misses and were also excluded from analysis [mean percent anticipations across tasks (SD) = 0. 41% (0. 74%); mean percent misses across tasks (SD) = 1.2% (2%)]. All correct trials with RTs between these lower and upper bounds were included in RT analyses. No further data cleaning or transformations of the data were applied prior to analysis^4^. For all analyses, the alpha level required for significance was set at *p* = 0.05 and, where appropriate, pairwise comparisons with Bonferroni correction were used to explore significant main effects and interactions. Greenhouse-Geisser corrected *p*-values are reported.

### Individual task analyses: Mean reaction time and accuracy

Table 3 presents mean reaction times and standard deviation of reaction times of performance for male and female participants on each level of the independent variables of interest for all tasks at the baseline and 24-h sessions. For each task, we analyzed task specific effects on reaction time across sessions using a series of mixed factor Analysis of Variance (ANOVAs), with a between-subjects factor of Sex (male, female) and within-subjects factors of Session (baseline, 24-h), and other independent variables related to the individual tests (described in Table 1). Only relevant task effects for reaction time are presented in text (below), but all main and interaction effect results for reaction time are presented in Table 3.

**Table 3:** **Reaction times (RT; in ms) for relevant variables on the DalCAB tasks for groups of male and female participants at the baseline and 24-h testing sessions**.

DalCAB Task variable^*^	**Male**		**Female**		**Main effects**	**Interactions**
	**Baseline Mean (SD)**	**24-h Mean (SD)**	**Baseline Mean (SD)**	**24-h Mean (SD)**		
**SRT**						
N	36	35	64	60	**RSI: *F*_(2, 186)_ = 206.88, *p* < .001**	RSI × Session: *F*_(2, 186)_ = 1.58, *p* = 0.209
Mean RT	252 (31)	255 (38)	274 (61)	265 (42)	Session: *F*_(1, 93)_ < 1, *p* = 0.745	RSI × Sex: *F*_(2, 186)_ < 1, *p* = 0.529
500 ms RSI^a^	282 (44)	288 (47)	300 (72)	296 (59)	Sex: *F*_(1, 93)_ = 3.14, *p* = 0.07	Session × Sex: *F*_(1, 94)_ = 2.27, *p* = 0.135
1000 ms RSI^a^	239 (32)	242 (48)	264 (60)	251 (39)		RSI × Session × Sex: *F*_(2, 186)_ < 1, *p* = 0.673
1500 ms RSI^a^	234 (28)	236 (28)	259 (57)	249 (36)		
Preparation effect^b^	−47 (31)	−52 (39)	−41 (34)	−48 (35)		
**GNG**						
N	36	35	64	61	**Percent-Go-trials:**	**Percent-Go-trials × Session: *F*_(1, 94)_ = 9.23,**
Mean RT: 20% Go	361 (72)	366 (90)	378 (83)	370 (82)	***F*_(1, 94)_ = 269.13, *p* < 0.001**	***p* = 0.003**
Mean RT: 80% Go	318 (74)	309 (79)	332 (82)	318 (87)	Session: *F*_(1, 94)_ = 1.21, *p* = 0.27	Percent-Go-trials × Sex: *F*_(1, 94)_ < 1, *p* = 0.67
					Sex: *F*_(1, 94)_ = 1.70, *p* = 0.19	Session × Sex: *F*_(1, 94)_ = 2.43, *p* = 0.122
						Percent-Go-trials × Session × Sex: *F*_(1, 94)_ < 1, *p* = 0.465
**CRT**						
N	36	35	64	61	**Trial type: *F*_(1, 94)_ = 108.34,**	Trial type × Session: *F*_(1, 94)_ < 1, *p* = 0.665
Mean RT	423 (130)	397 (121)	435 (125)	409 (117)	***p* < 0.001**	Trial type × Sex: *F*_(1, 94)_ < 1, *p* = 0.607
No Switch	412 (71)	383 (57)	420 (57)	393 (53)	**Session: *F*_(1, 94)_ = 23.94,**	Session × Sex: *F*_(1, 94)_ < 1, *p* = 0.76
Switch	445 (64)	417 (52)	449 (57)	426 (63)	***p* < 0.001**	Trial type × Session × Sex: *F*_(1, 94)_ < 1, *p* = 0.503
Switch Cost^c^	34 (42)	33 (32)	29 (32)	33 (33)	Sex: *F*_(1, 94)_ < 1, *p* = 0.45	
**DUAL TASK CRT**						
N	36	29	64	59	**Trial type: *F*_(1, 86)_ = 583.16,**	Trial type × Session: *F*_(1, 86)_ = 2.64, *p* = 0.108
Mean RT	529 (87)	482 (75)	534 (90)	473 (68)	***p* < 0.001**	Trial type × Sex: *F*_(1, 86)_ < 1, *p* = 0.724
No Switch	463 (81)	419 (69)	464 (72)	410 (54)	**Session: *F*_(1, 86)_ = 64.33,**	Session × Sex: *F*_(1, 86)_ = 1.28, *p* = 0.26
Switch	610 (102)	560 (92)	619 (115)	550 (87)	***p* < 0.001**	Trial type × Session × Sex:
Switch Cost^c^	146 (54)	141 (50)	155 (67)	140 (54)	Sex: *F*_(1, 86)_ < 1, *p* = 0.89	*F*_(1, 86)_ = 1.16, *p* = 0.28
WM load effect^d^	101 (88)	85 (55)	99 (63)	65 (61)		
**FLANKER**						
N	36	35	63	61	**FC^g^: *F*_(1, 94)_ = 105.11, *p* < 0.001**	FC^g^ × Session: *F*_(1, 94)_ < 1, *p* = 0.784
Mean RT	455 (107)	444 (118)	484 (122)	468 (122)	**Session: *F*_(1, 94)_ = 9.61,**	FC^g^ × Sex: *F*_(1, 94)_ < 1, *p* = 0.501
Congruent	445 (48)	435 (54)	476 (59)	459 (65)	***p* = 0.003**	Session × Sex: *F*_(1, 94)_ = 1.15, *p* = 0.28
Incongruent	464 (47)	453 (48)	492 (57)	475 (61)	**Sex: *F*_(1, 94)_ = 6.29, *p* = 0.014**	FC^g^ × Session × Sex: *F*_(1, 94)_ < 1, *p* = 0.783
Interference effect^e^	19 (24)	18 (22)	16 (20)	16 (19)		
**ITEM WORKING MEMORY**						
N	23	23	43	42	**Set size: *F*_(2, 126)_ = 220.24,**	**Set size × target: *F*_(2, 126)_ = 3.64, *p* = 0.034**
Mean RT	828 (286)	788 (284)	830 (247)	765 (241)	***p* < 0.001**	Set size × session: *F*_(2, 126)_ < 1, *p* = 0.749
Set size: 2	726 (136)	666 (96)	731 (98)	682 (106)	Target: *F*_(1, 63)_ = 1.93, *p* = 0.169	Set size × sex: *F*_(2, 126)_ = 1.45, *p* = 0.239
Set size: 4	852 (126)	813 (147)	860 (117)	793 (104)	**Session: *F*_(1, 63)_ = 27.72,**	Target × session: *F*_(1, 63)_ = 1.25, *p* = 0.267
Set size: 6	937 (166)	892 (185)	929 (117)	852 (133)	***p* < 0.001**	Target × sex: *F*_(1, 63)_ < 1, *p* = 0.819
Set-size slope^f^	113 (61)	117 (70)	103 (39)	84 (44)	Sex: *F*_(1, 63)_ < 1, *p* = 0.789	Session × sex: *F*_(1, 63)_ < 1, *p* = 0.479
						Session × target × sex: : *F*_(1, 63)_ < 1, *p* = 0.458
						Session × set size × sex: *F*_(2, 126)_ = 1.75,
						*p* = 0.178
						Target × set size × sex: *F*_(2, 126)_ < 1, *p* = 0.63
						Session × target × set size: *F*_(2, 126)_ < 1,
						*p* = 0.839
						Set size × target × session × sex:
						*F*_(2, 126)_ = 2.55, *p* = 0.087
**VISUAL SEARCH**						
N	36	35	64	61		
**Feature Search**						
Mean RT	624 (178)	593 (181)	660 (238)	621 (229)	**Set size: *F*_(2, 188)_ = 7.46,**	Set size × Session: *F*_(2, 188)_ = 2.00, *p* = 0.138
Set size: 6	616 (63)	584 (65)	649 (93)	613 (84)	***p* < 0.001**	Set size × Sex: *F*_(2, 188)_ < 1, *p* = 0.859
Set size: 12	631 (78)	591 (69)	667 (140)	619 (81)	**Session: *F*_(1, 94)_ = 17.44,**	Session × Sex: *F*_(1, 94)_ < 1, *p* = 0.487
Set size: 18	625 (70)	602 (72)	665 (140)	630 (112)	***p* < 0.001**	Set size × Session × Sex: *F*_(2, 188)_ < 1,
Set-size slope^f^	< 1 (3)	2 (2)	1 (6.0)	1 (5.3)	Sex: *F*_(1, 94)_ = 3.53, *p* = 0.064	*p* = 0.818
**Conjunction Search**						
Mean RT	1319 (783)	1123 (575)	1281 (677)	1139 (594)	**Set size: *F*_(2, 188)_ = 639.02,**	**Set size × Session: *F*_(2, 188)_ = 13.77,**
Set size: 6	1006 (138)	884 (123)	1007 (188)	912 (148)	***p* < 0.001**	***p* < 0.001**
Set size: 12	1328 (220)	1123 (150)	1281 (229)	1130 (212)	**Session: *F*_(1, 94)_ = 128.68,**	Set size × Sex: *F*_(2, 188)_ < 1, *p* = 0.357
Set size: 18	1613 (335)	1352 (160)	1557 (305)	1368 (276)	***p* < 0.001**	Session × Sex: *F*_(1, 94)_ = 2.65, *p* = 0.107
Set size slope^f^	51 (23)	39 (11)	46 (19)	38 (14)	Sex: *F*_(1, 94)_ < 1, *p* = 0.857	Set size × Session × Sex: *F*_(2, 188)_ < 1,
						*p* = 0.536

* *Please refer to written results section for a description of the effects of DalCAB task variables on mean reaction time*.

a *Response-stimulus interval*.

b *Preparation effect = 1500 – 500 ms RSI mean RT*.

c *Switch cost = mean RT switch trials - mean RT non-switch trials*.

d *Working memory load effect = dual task mean RT - CRT mean RT*.

e *Interference effect = incongruent mean RT - congruent mean RT*.

f *Slope units = ms/item*.

g *Flanker congruency*.

*Bold indicates significance at the p < 0.05*.

Table 4 presents mean accuracy and standard deviation of accuracy of performance for male and female participants on each level of the independent variables of interest for all tasks at the baseline and 24-h sessions. With the exception of the choice reaction time component of the dual task (described below) and the go-no-go task, a comparison of the pattern of mean RTs and accuracy within each task revealed no apparent speed-accuracy trade-offs. Given that the DalCAB was designed to assess attentional functioning using predominantly RT measures, and accuracy was high in our young, healthy adult sample (see Table 4), we have only presented analysis of accuracy data for a few relevant measures below (percent false alarms in the Go/No-Go task and percent correct in the item work memory task). All main and interaction effects on accuracy in the Go/No-Go and item working memory tasks are presented in Table 4. Standard error of the mean (SE) of RT and accuracy data are provided in the text where appropriate.

**Table 4:** **Mean accuracy for independent variables on the DalCAB tasks for groups of male and female participants at the baseline and 24-h testing sessions**.

DalCAB Task variable^*^	**Male**		**Female**		**Main effects**	**Interactions**
	**Baseline Mean (SD)**	**24-h Mean (SD)**	**Baseline Mean (SD)**	**24-h Mean (SD)**		
**SRT**						
N	36	35	64	60		
Mean % correct	98.1 (2.4)	95.8 (4.6)	98.8 (1.5)	97.9 (2.2)		
500ms RSI^a^	99.9 (0.1)	98.9 (4.6)	99.8 (4.5)	99.7 (2.0)		
1000ms RSI^a^	99.0 (2.0)	98.1 (3.2)	98.8 (3.0)	98.8 (2.1)		
1500ms RSI^a^	95.6 (6.6)	90.4 (8.6)	97.7 (3.7)	95.3 (5.3)		
**GNG**						
N	36	35	64	61	*PC*	*PC*
					Percent go-trials: *F*_(1, 94)_ < 1,	Percent-Go-trials × Session: *F*_(1, 94)_ < 1,
20% Go: % correct	99.7 (1.7)	99.4 (2.4)	100 (0)	99.5 (2.2)	*p* = 0.669	*p* = 0.97
					**Session: *F*_(1, 94)_ = 5.19,**	Percent-Go-trials × Sex: *F*_(1, 94)_ < 1,
20 % Go: % FA^b^	0.3 (0.6)	0.3 (0.7)	0.3 (0.6)	0.2 (0.5)	***p* = 0.025**	*p* = 0.77
80% Go: % correct	99.8 (0.5)	99.3 (2.2)	99.9 (.3)	99.8 (1.2)	Sex: *F*_(1, 94)_= 1.7, *p* = 0.19	Session × Sex: *F*_(1, 94)_ < 1, *p* = 0.75
	8.1 (9.3)	8.9 (6.8)	6.6 (6.8)	10.2 (9.7)	*FA*	Percent-Go-trials × Session × Sex:
80 % Go: % FA^b^					**Percent go-trials:**	*F*_(1, 94)_ = 2.87, *p* = 0.093
					***F*_(1, 94)_ = 129.93, *p* < 0.001**	*FA*
					**Session: *F*_(1, 94)_ = 4.32, *p* = 0.04**	**Percent-Go-trials** × **Session:**
					Sex: *F*_(1, 94)_ = 1.10, *p* = 0.29	***F*_(1, 94)_ = 4.98, *p* = 0.031**
						Percent-Go-trials × Sex: *F*_(1, 94)_ < 1,
						*p* = 0.986
						Session × Sex: *F*_(1, 94)_ < 1, *p* = 0.75
						Percent-Go-trials × Session × Sex:
						*F*_(1, 94)_ = 2.0, *p* = 0.16 *F*_(1, 94)_ = 2.0,
						*p* = 0.16
**CRT**						
N	36	35	64	61		
Mean % correct	95.0 (5.7)	95.0 (4.4)	96.4 (2.8)	96.4 (3.2)		
No Switch	95.0 (6.8)	95.3 (5.4)	96.2 (3.3)	96.3 (4.1)		
Switch	95.1 (5.8)	94.6 (4.9)	96.6 (3.3)	96.4 (3.6)		
**DUAL TASK CRT**						
N	36	29	64	59		
Mean % correct	96.7 (3.0)	96.1 (3.9)	97.2 (3.2)	96.4 (3.3)		
No Switch	98.1 (2.7)	97.6 (3.1)	98.0 (3.4)	97.4 (2.7)		
Switch	95.1 (4.0)	94.2 (5.2)	96.1 (3.8)	95.1 (4.6)		
Interference effect^c^	1.7 (4.5)	1.5 (3.5)	0.8 (3.4)	−0.1 (2.7)		
**FLANKER**						
N	36	35	63	61		
Mean % correct	94.5 (4.1)	94.5 (4.2)	95.1 (4.4)	95.8 (4.1)		
Congruent	94.8 (4.1)	95.3 (3.8)	95.7 (4.7)	96.5 (4.3)		
Incongruent	94.3 (5.1)	93.7 (5.5)	94.4 (5.2)	95.3 (4.8)		
**ITEM WORKING MEMORY**						
N	23	23	43	42	**Set size: *F*_(2, 126)_ = 121.86,**	**Set size × target: *F*_(2, 126)_ = 10.87,**
Mean % correct	81.9 (6.0)	83.2 (8.1)	79.9 (7.9)	82.8 (7.0)	***p* < 0.001**	***p* < 0.001**
Set size: 2	93.5 (5.1)	90.4 (8.2)	90.1 (9.3)	92.4 (7.7)	**Target: *F*_(1, 63)_ = 15.64, *p* < 0.001**	Set size × session: *F*_(2, 126)_ = 2.27,
Set size: 4	79.4 (7.9)	85.9 (8.1)	79.8 (12.3)	82.1 (11.6)	**Session:*F*_(1, 63)_ = 5.44, *p* = 0.023**	*p* = 0.109
Set size: 6	73.0 (12.2)	77.6 (12.0)	70.1 (11.9)	71.0 (10.3)	Sex: *F*_(1, 63)_ = 2.11, *p* = 0.15	Set size × sex: *F*_(2, 126)_ = 1.59, *p* = 0.211
						Target × session: *F*_(1, 63)_ = 1.33, *p* = 0.253
						Target × sex: *F*_(1, 63)_ = 2.19, *p* = 0.144
						Session × sex: *F*_(1, 63)_ < 1, *p* = 0.642
						Session × target × sex: *F*_(1, 63)_ = 1.22,
						*p* = 0.274
						**Session × set size × sex:** ***F*_(2, 126)_ = 3.33, *p* = 0.04** **Target × set size × sex:** ***F*_(2, 126)_ = 3.48,** ***p* = 0.035** Session × target × set size: *F*_(2, 126)_ < 1, *p* = 0.939 Set size × target × session × sex: *F*_(2, 126)_ = 1.21, *p* = 0.301
**VISUAL SEARCH**						
N	36	35	64	61		
**Feature search**						
% correct	94.3 (3.6)	93.7 (6.6)	94.1 (5.0)	94.6 (4.2)		
Set size: 6	94.2 (4.7)	93.4 (5.8)	94.1 (6.1)	94.3 (5.8)		
Set size: 12	94.1 (5.0)	93.4 (7.0)	93.6 (5.9)	94.8 (5.6)		
Set size: 18	94.5 (4.7)	94.1 (8.6)	94.5 (5.8)	94.7 (4.3)		
**Conjunction search**						
% correct	95.6 (5.4)	94.6 (6.3)	94.8 (5.7)	95.6 (4.0)		
Set size: 6	95.9 (5.1)	93.8 (7.1)	94.9 (6.7)	95.5 (4.5)		
Set size: 12	95.1 (6.5)	95.4 (5.6)	95.2 (6.4)	95.5 (5.9)		
Set size: 18	95.7 (6.6)	94.7 (8.8)	94.3 (6.1)	95.9 (4.6)		

* *Please refer to the written results section for a description of select DalCAB task variables on accuracy*.

a *Response-stimulus interval*.

b *False alarm*.

c *Interference effect = dual task mean % correct − CRT mean % correct*.

*Bold indicates significance at the p < 0.05*.

### Individual task performance analyses: Practice effects

To quantify the change in reaction time across the repeated testing sessions, mean practice effects (24-h mean—baseline mean) for overall mean RT and mean RT across the levels of the DalCAB task variables of interest are presented in Table 5. Differences in reaction times between baseline and the 24-h session were assessed using paired samples *t*-tests. Standard deviation of the difference scores in performance and effect sizes for practice effects (Cohen's d) were also calculated for the session 24-h after baseline. These values are also shown in Table 5. The ICC scores, practice effects, standard deviation of the difference score, and effect sizes can be used to compute reliable change indices.

**Table 5:** **Change-based reliability statistics for participants 24-h after baseline**.

**DalCAB Task**	**24-h**			
	**ICC**	**PE**	**SD_D_**	**Cohen's d**
**SRT**				
N	95			
Mean RT	**0.825**^#^	−2.7 ms	35.6 ms	0.07
RT preparation effect^a^	0.570^#^	−6.1 ms	37.8 ms	0.16
**GNG**				
N	96			
20% Go RT	**0.836**^#^	0.4 ms	35.8 ms	0.01
20 % Go: % FA^b^	0.252	−0.1%	0.8%	0.07
80% Go RT	**0.847**^#^	−9.7 ms*	31.2 ms	0.32
80 % Go: % FA^b^	0.548^#^	2.4%^*^	9.2%	0.27
**CRT**				
N	96			
Mean RT	**0.749**^#^	−24.0 ms^*^	46.8 ms	**0.51**
RT switch cost^c^	0.609^#^	2.4 ms	36.6 ms	0.07
**DUAL TASK CRT**				
N	88			
Mean RT	**0.781**^#^	−50.4 ms^*^	53.0 ms	**0.99**
RT switch cost^c^	**0.817**^#^	−10.0 ms^*^	44.3 ms	0.23
WM load RT effect^d^	**0.735**^#^	−26.0 ms^*^	54.2 ms	0.48
DT Interference effect^e^	0.496^#^	−0.7%	3.7%	0.18
**FLANKER**				
N	96			
Mean RT	**0.871**^#^	−13.2 ms^*^	36.6 ms	0.36
RT Interference effect^f^	0.301	−0.7 ms	26.7 ms	0.02
**ITEM MEMORY**				
N	65			
Mean RT	**0.795**^#^	−57.8 ms^*^	81.1 ms	**0.71**
Set-size RT slope	0.623^#^	−3.5 ms	26.4 ms	0.14
% correct	0.417	2.5%^*^	8.7%	0.29
**VISUAL SEARCH**				
N	96			
**Feature search**				
Mean RT	**0.770**^#^	−35.9 ms^*^	77.4 ms	0.49
Set-size RT slope	0.096^#^	0.30 ms	6.70 ms	0.06
**Conjunction search**				
Mean RT	**0.718**^#^	−163.2 ms^*^	142.4 ms	**1.19**
Set-size RT slope	0.464^#^	−9.29 ms^*^	19.91	**0.53**

Bold numbers highlight good to very good reliability and significantly large practice effects. ICC, Intraclass correlation (two-way random, absolute agreement, average measures);

# , significant F-test (p < 0.01); Practice effect (PE), post session mean—baseline mean;

* *significant difference based on paired t-test (p < 0.05); SD_D_, standard deviation of the difference score*.

a *Preparation effect = 1500 – 500 ms RSI RT*.

b *False alarm*.

c *Switch cost = RT Switch - RT No-switch*.

d *Working memory load RT effect = dual task mean minus CRT mean*.

e *DT interference effect = dual task CRT accuracy – CRT accuracy*.

f *Interference effect = incongruent RT − congruent RT*.

### Individual task performance analysis: Test-retest reliability

The test-retest reliability of performance as defined by the dependent variables in each task was analyzed using intra-class correlations (ICC) comparing the 24-h session data to baseline. ICC are presented in Table 5. For the current analysis, intraclass correlations at or above 0.700 were considered “Good,” 0.800 or higher were considered “Very Good” and 0.900 or higher were considered “Excellent” (Dikmen et al., 1999; Van Ness et al., 2008; Chen et al., 2009).

## Results

Overall, the expected pattern of effects across the independent variables of interest within each task were found (as described in Table 1) and the majority of the ICCs (11 of 19) were greater than 0.700 (i.e., at least *Good* reliability). A specific description of these results within each task is presented below.

### Simple reaction time (SRT)

One female participant was removed from the SRT task analysis due to an extremely low percentage of correct responses in the 24-h session (61% missed trials, i.e., RTs > 1500 ms). The mixed factor ANOVA on mean reaction time (as described above), including the task variable of RSI, revealed the anticipated preparation effect; i.e., faster responses at longer RSIs [M_RT ± SE_ RSI500 = 290.48 ± 5.65 > M_RT ± SE_ RSI1000 = 247.77 ± 4.31 (*p* < 0.001) > M_RT ± SE_ RSI1500 = 243.60 ± 3.92 (*p* = 0.04); *F*_(2, 186)_ = 206.88, *MSE* = 792.39, Table 3]. Twenty-four hour practice effects were not significant for the SRT task, for overall mean reaction time or the preparation effect (Table 5).

The test-retest reliability (ICC) score of mean RTs in the 24-h session was *Very Good* (0.825). In contrast, ICC scores for the RT preparation effect (24 h session RT – baseline RT) were statistically significant but much lower, falling below the *Good* range (0.570).

### GO/NO-GO (GNG)

The mixed factor ANOVA on mean reaction time and accuracy (as described above) including the task variable of percent-Go-trials (20%-Go, 80%-Go) revealed the expected pattern of faster reaction times [*F*_(1, 94)_ = 269.13, *MSE* = 784.88, *p* < 0.001] and more false alarms [FA; *F*_(1, 94)_ = 129.93, *MSE* = 0.005, *p* < 0.001] in the 80%-Go condition than the 20%-Go condition at baseline and 24-h later (Tables 3, 4; there were no misses in the Go-no-go task). Practice effects for reaction times and false alarms were significant at 24-h for the 80% Go-trial condition only (Table 5) and revealed a possible speed-accuracy tradeoff in the Go/No-Go task; participants responded faster and made more false alarms overall at 24-h compared to baseline (M_RT ± SE_ 24-h = 315.22 ± 4.81, M_RT ± SE_ baseline = 324.94 ± 4.17, *p* = 0.003; M_FA ± SE_ 24-h = 9.7 ± 0.9%, M_FA ± SE_ baseline = 7.2 ± 0.8%, *p* = 0.011).

ICC scores of mean RTs for 20 and 80%-Go trials at the 24-h session were in the *Very Good* range (24-h: 0.836 and 0.847, respectively). In contrast, the reliability score for the percent false alarms was below the *Good* range for both the 20 and 80% Go trial conditions at the 24-h interval (ICC = 0.252 and 0.548, respectively).

### 2-choice reaction time (CRT)

The mixed factor ANOVA on mean reaction time (as described above), including the task variable of Trial Type (switch, non-switch) revealed the expected pattern of switch costs; participants were slower to respond when the stimulus/response changed on consecutive trials (i.e., M_RT ± SE switch trials_ = 432.9 ± 5.6 ms) than when the stimulus/response remained the same [i.e., M_RT ± SE non−switch trials_ = 400.6 ± 5.6 ms; *F*_(1, 94)_ = 108.34, *MSE* = 857.65, *p* < 0.001]. A significant practice effect for mean RT was found indicating that participants were faster at 24-h than at baseline (M_RT 24−h ± SE_ = 404.6 ± 5.7 ms, M_RT baseline ± SE_ = 428.9 ± 6.1 ms, *p* < 0.001); no practice effects were found for the RT switch cost (Table 5).

The test-retest reliability score of mean RTs at the 24-h interval was in the *Good* range (24-h: 0.749), but the test-retest reliability score for the RT switch cost (i.e., RT Switch minus RT No-switch) was below the *Good* range (24-h: 0.609, Table 5).

### Dual task

Eight participants (2 women, 6 men) were removed from the dual task CRT analysis due to a complete lack of responding to CRT trials in the 24-h session. Thus, 58 women and 30 men were included in the mixed factor dual task CRT analysis. The mixed factor ANOVA on mean RT (as described above), including the task variable of Trial Type (switch, non-switch) revealed the expected pattern of switch costs; participants were slower to respond when the stimulus/response changed on consecutive trials (i.e., M_RT ± SE switch trials_ = 581.4 ± 10.7 ms) than when the stimulus/response remained the same [i.e., M_RT ± SE non−switch trials_ = 436.6 ± 6.69 ms; *F*_(1, 86)_ = 583.16, *MSE* = 2796.3, *p* < 0.001, Table 3]. A significant practice effect for mean RT indicated that participants were significantly faster at 24-h than at baseline (M_RT baseline ± SE_ = 539.5 ± 8.8 ms, M_RT 24−h ± SE_ = 483.38 ± 7.5 ms, *p* < 0.001). This improvement in performance RT between baseline and 24-h was paired with a decrease in performance accuracy (percent correct), suggesting a speed-accuracy trade-off (M_PC baseline ± SE_ = 97 ± 0.32 %, M_PC 24−h ± SE_ = 96 ± 0.37 %, *p* < 0.001, **not presented in table**). The RT switch cost and WM load RT effect were also significantly reduced 24-h after baseline (M_RT switch cost baseline ± SE_ = 152.19 ± 6.2 ms, M_RT switch cost 24−h ± SE_ = 140.52 ± 5.6 ms, *p* = 0.037; M_WM load RT baseline ± SE_ = 107.18 ± 7.2, M_WM load RT 24−h ± SE_ = 79.34 ± 6.2 ms, *p* < 0.001; Table 5). No significant practice effects were found for the DT interference effect (Table 5) indicating that the additional workload from adding a secondary task to the choice reaction time task did not change across testing sessions.

The ICC scores for mean RTs and the RT switch cost at 24-h interval were in the *Good* to *Very Good* range (Mean RT: 0.781 and RT switch cost: 0.817; Table 5). The test-retest reliability score for the WM load RT effect (difference in reaction time performance between the dual tasks and CRT) 24-h after baseline was *Good* (0.735), but the dual task interference effect (difference in accuracy between dual task CRT and CRT) was below the *Good* range (0.496).

### Flanker

The mixed factor ANOVA on mean reaction time including the task variable of Flanker Congruency (congruent, incongruent) revealed that RTs were faster for congruent trials than incongruent trials [congruent = 452.8 ± 5.6 ms, incongruent = 470.2 ± 5.5 ms; *F*_(1, 94)_ = 105.11, *MSE* = 256.05, *p* < 0.001]. RTs for male performance were also faster than RTs for female performance [males = 447.6 ± 8.8 ms, females = 475.4 ± 6.7 ms; *F*_(1, 94)_ = 6.29, *MSE* = 10903.81, *p* = 0.014, Table 3]. A significant practice effect for mean RT was found; participants were faster at 24-h than at baseline (M_RT 24−h ± SE_ = 458.74 ± 6.01 ms, M_RT baseline ± SE_ = 471.90 ± 5.55 ms, *p* < 0.05, Tables 3, 5). The RT interference effect did not differ across testing sessions (Table 5).

The test-retest reliability score of mean RTs at the 24-h session was *Very Good* (0.871). However, the test-retest reliability scores for the RT interference effect (i.e., incongruent RT minus congruent RT) was less than *Good* (0.301, Table 5).

### Item working memory

Due to changes in programming, the final version of the item working memory task was not completed by all participants (*n* = 65; 42 female). The mixed factor ANOVA on mean reaction time including the task variables of Set Size (2, 4, 6) and Target (present, absent) revealed the anticipated effect of Set Size, such that RTs were significantly slowed for each additional two-item increase in working memory set size [*F*_(2, 126)_ = 220.24, *MSE* = 11292.38, *p* < 0.001; Set 2 = 701.4 ± 11.8 ms, Set 4 = 830.0 ± 13.4 ms, Set 6 = 904.5 ± 15.7 ms, all *p* < 0.001] and accuracy (percent correct) significantly decreased for each additional two-item increase in working memory set size (Set 2 = 92 ± 0.8%, Set 4 = 82 ± 11%, Set 6 = 73 ± 12%, all *p* < 0.001). A significant interaction between Set size and Target was also found for reaction time [*F*_(2, 126)_ = 3.64, *MSE* = 8039.09, *p* = 0.03]; *post-hoc* pairwise comparisons indicated the same pattern of effect of Set size on RTs for both target absent and target present trial types (all *p* < 0.01; mean RT is presented collapsed across target in Tables 3, 4). Two significant three way interactions for percent correct were also found. An interaction among Set size, Target and Sex [*F*_(2, 126)_ = 3.48, *p* = 0.035] revealed the above reported decrease in percent correct performance across working memory set size increase for women, regardless of Target. In contrast, for men, a decrease in accuracy was found between Set 2 and Set 6 for both Target types, but only between Set 4 and Set 6 when the Target was *present* in the working memory set. (Target Absent: Set 2: 93%, Set 4: 75%, Set 6: 69%; Target Present: Set 2: 91%, Set 4: 90%, Set 6: 82%). Similarly, the interaction between Session, Set size and Sex [*F*_(2, 126)_ = 3.33, *p* = 0.04] revealed the above described decrease in accuracy across Set size for women, regardless of session, but not for men (Session 1: Set 2 > Set 4, *p* < 0.001; Set 2 > Set 6, *p* < 0.001; Set 4 = Set 6, *p* = 0.164; Session 2: Set 2 = Set 4, *p* = 1.0; Set 2 > Set 6, *p* = 0.007; Set 4 > Set 6, *p* = 0.006; mean percent correct is presented collapsed across target type in Table 4).

A significant practice effect for mean RT indicated that participants were faster at 24-h than at baseline (M_RT 24−h ± SE_ = 780.77 ± 14.31 ms, M_RT baseline ± SE_ = 838.54 ± 13.77 ms, *p* < 0.001); participants were also more accurate at 24-h than at baseline (Table 5). The practice effect for the set size RT slope was not significant.

The test-retest reliability score for mean RTs at the 24-h session was in the *Good* range (0.795), whereas the ICC scores for the set size slope and mean percent of correct responses (M_PC_) fell below the *Good* range (0.623 and 0.417, respectively).

### Visual search

Mixed factor ANOVAs on mean reaction time including the task variable of Set Size (6, 12, and 18) were performed for each Search Type (feature, conjunction) separately.

In the feature search task, a significant main effect of Set Size was found; RTs were faster for the smallest set than the two larger set sizes, which did not differ [*F*_(2, 188)_ = 7.46, *MSE* = 1504.13, *p* < 0.001; Set 6 = 619.4 ± 8.8 ms, Set 12 = 630.9 ± 10.1 ms, Set 18 = 634.4 ± 11.0 ms; Set 6 < Set 12, *p* = 0.008; Set 6 < Set 18, *p* = 0.004; Set 12 = Set 18, *p* = 1.0]. While the set size main effect was significant, it should be noted that search slopes were small and almost 0 (range from < 1 to 2 ms/item), in line with other feature search tasks (reviewed in Wolfe, 1998). A significant practice effect for mean RT was found in the feature search task; participants' mean RT was faster at 24-h than at baseline (M_RT 24−h ± SE_ = 610.25 ± 8.22 ms, M_RT baseline ± SE_ = 646.16 ± 10.96 ms, *p* < 0.001), but there was no change in the set size slope. The ICC score for mean RTs was in the *Good* range (0.770, Table 5).

In the conjunction search task, a significant main effect of Set Size was also found (slopes ranging from 46 to 51 ms/item), although qualified by a significant Session × Set Size interaction [*F*_(2, 188)_ = 13.77, *MSE* = 1565.64, *p* < 0.001]. *Post-hoc* analysis of the interaction showed that while RTs increased significantly as a function of set size in each session (i.e., the anticipated set size effect) and RTs were faster at the 24-h session than the baseline session for each set size (consistent with practice effects, Table 5), the set size slope decreased between baseline and the 24-h session. This interaction reflects the significant practice effects found in the conjunction search task for mean RT and set-size slope; participants were significantly faster overall and showed a smaller set size slope at 24-h than at baseline (M_RT 24−h ± SE_ = 1130.5 ± 18.2 ms, M_RT baseline ± SE_ = 1293.7 ± 22.4 ms, *p* < 0.001). The ICC score for mean RTs for the conjunction visual search task was also in the *Good* range (0.718, Table 5).

## Discussion

The current study presents a unique look at average performance (reaction time, accuracy), 24-h practice effects and reliability data for seven common attention test paradigms integrated as sub-tests of the DalCAB in a sample of 96 healthy adults (18–31 years of age). These results provide the first normative data for the DalCAB and present preliminary information on the psychometric properties of performance on several tasks previously used in the cognitive neuroscience field to assess separate aspects of attention^5^. These tasks are combined within the DalCAB which employs standardized stimuli across all tasks, allowing comparisons across attention functions using multiple test measures.

### Test-retest reliability

Our primary goal was to examine the stability of performance on tasks of the DalCAB by examining the test-retest reliability of the DalCAB outcome measures over a short period of time (24 h). Test-retest reliability in performance is an important property to consider in cognitive or clinical neuroscience research, particularly with repeated testing paradigms or for evaluating change in performance patterns over time. At a re-test interval of 24 h, we found that all reliability coefficients for mean raw RT scores on the individual DalCAB subtests demonstrated *Good* to *Very Good* test-retest reliability, ranging from 0.718 to 0.871. These values are similar to or better than reliability findings on RT tasks from other batteries by young and older subjects at a variety of test-retest intervals (e.g., Secker et al., 2004; Falleti et al., 2006, Table 1; Williams et al., 2005, Table 3; Lowe and Rabbitt, 1998; Nakayama et al., 2014).

In contrast, although the Dual Task CRT switch cost maintained *Very Good* reliability and the Dual Task Working Memory Load Effect was above 0.700 at the 24-h interval, in general, the test-retest reliability of RT-difference measures (e.g., SRT preparation effect, CRT switch cost, Flanker interference effect) tended to be less robust than the reliability of mean performance RT.

The pattern of lower test-retest reliability for difference performance measures on the DalCAB is similar to that reported for the ANT (Fan et al., 2002; MacLeod et al., 2010). For example, MacLeod et al. (2010), using data derived from a large sample of 15 studies, reported low split-half reliability for the vigilance and orienting difference measures computed from the ANT (0.38 and 0.55, respectively) while the executive score reliability was higher (0.81). Assessments of the test-retest reliability of the ANT and ANT-I have also revealed variations in the reliability of the three difference scores used to describe attention network efficiency, with the executive control score reported as the most reliable and the vigilance score reported as the least reliable (Fan et al., 2002; Ishigami and Klein, 2010; Ishigami et al., 2016). Due to the nature of correlation analysis, test-retest reliability findings obtained from calculation of difference scores that are highly correlated could be less reliable (discussed in MacLeod et al., 2010). These findings should lend some caution to the use of difference scores as sole measures of attentional function. The reliability of the DalCAB is also lower than the ANT reliability overall, likely due to the fact that the ANT has more trials.

Test-retest reliability of performance accuracy was also analyzed on all tasks. However, given our sample of healthy adults (18–31 years of age), accuracy was high across all administrations of the DalCAB (as presented in Table 4) and thus we presented performance accuracy results in the Go/No-Go and Item working memory tasks only (Table 5). Test-retest reliability coefficients for the Go/No-Go and Item working memory performance accuracy measures were lower than those observed for performance RT, perhaps due to the low variance in these measures given our sample (see also a discussion by Falleti et al., 2006 about ceiling and floor effects). For example, in the Go/No-Go task, false alarms in the 20% go frequency task were very low, ranging from 0.2 to 0.6 % and thus reliability was also very low (0.252). In contrast, the frequency of false alarms in the 80% go frequency task ranged from 6.6 to 12.9% and thus test-retest reliability was higher (0.548). We also found that reliability of percent correct in the Item Working Memory task of the DalCAB also fell well below our definition of *Good* (0.417) at the 24-h interval.

Hahn et al. (2011, Table 2) reported ICCs falling below 0.700 in healthy adults (Mean age = 33 years) for the alerting (0.166) and orienting (0.109) accuracy measures, as well as the overall mean accuracy measure (0.553) on the ANT (Fan et al., 2002). Similarly, using the Continuous Performance Test-II (Conners, 2000), Zabel et al. (2009) reported test-retest reliability scores of 0.39 and 0.57 on omission and commission errors, respectively, in a sample of healthy children (6–18 years); these ICCs were less than that reported for reaction times on hit trials (ICC = 0.65). In contrast, Wöstmann et al. (2013, Table 2) reported an ICC of 0.84 for commission errors in the No-go portion of a Go/No-go task approximately 28 days following baseline, suggesting *Very Good* (0.800) test-retest reliability for this measure (in healthy 18–55 year olds). These authors also reported ICCs of 0.51 and 0.48 for accuracy of responses to number and shape stimuli in a continuous performance test (participants were instructed to remove their finger from a button when identical consecutive stimuli were presented; Wöstmann et al., 2013, Table 2). As accuracy measures, particularly those measured in healthy populations, tend to approach ceiling, it is possible that the Go/No-go and Item working memory performance accuracy measures with our current sample are not sufficiently varied to provide a representative account of test-retest reliability. This could be one reason why the ICCs for accuracy measures are often reported to be lower overall than the corresponding RT scores (e.g., Llorente et al., 2001; Messinis et al., 2007; Zabel et al., 2009; Hahn et al., 2011; Wöstmann et al., 2013). In the case of the DalCAB assessment presented here, participants were highly accurate in all presentations of both the Go/No-go and Item working memory tasks.

Overall, we found *Good* to *Very Good* test-retest reliability (ICC range: 0.718 to 0.871) on all of our mean reaction time measures for each of the DalCAB tasks, suggesting that the mean reaction time performance measures in all DalCAB tasks would allow for measurement of attention across short duration (24 h) repeated testing sessions. We also found much lower test-retest reliability (less than *Good*) for computed reaction time difference scores (ICC range: 0.096–0.62) and accuracy measures (ICC range: 0.25–0.548) indicating the lack of reliability these measures could offer across repeated testing sessions.

### Practice effects

While significant practice effects on computerized tests of attention and executive functions are common across various time periods (minutes, hours, and weeks), the practice effects on computerized batteries have tended to be largest between the first and second assessment and then remain stable over further presentations (Collie et al., 2003; Falleti et al., 2006). The DalCAB data revealed large and significant practice effects at the 24-h re-test interval on most tasks. Our results illustrate that participants were faster to respond in the repeat 24-h session than in the baseline session for all tasks except Simple Reaction Time and Go/No-Go: 20%-Go. In addition, although accuracy did not generally differ across sessions for the tasks, there were more anticipations (i.e., decreased accuracy) in the Simple Reaction Time task in the 24-h session than at baseline (Table 4). The lack of practice effects and decreased accuracy in performance on these basic alertness/vigilance tasks could suggest that the healthy adults maintained alertness and maximized their speed on both testing occasions, but were not as engaged and responded more haphazardly on repeated exposure to the SRT task; i.e., more transient arousal changes when the task was no longer novel (Steinborn and Langner, 2012). In previous research, while many behavioral measures exhibit practice effects, those requiring problem-solving and strategy use tend to show the greatest practice effects (Lezak, 1995; Dikmen et al., 1999). Thus, the practice effects apparent on more complex DalCAB tasks are likely related to improvements in processes rather than basic speed of responding, which was apparently maximized in both sessions.

In the dual task CRT trials, participants were faster and less accurate in the 24-h session than at baseline (Tables 3, 4). It should be noted that, in the 24-h session, eight participants failed to make responses to the CRT-trials while they completed the concurrent color-counting task of the dual task and others were prompted by the experimenter to make a button-press to each stimulus presented—these participants were not included in the analysis of the dual task. Regardless, the higher number of missed/incorrect responses and related decrease in percent correct CRT responses in the current data may reflect interference by the color-counting task; i.e., the color-counting task is so resource intensive for some participants that the concurrent CRT task is stopped completely. Thus, it is possible that there are two separate processes measurable by the DalCAB Dual Task: a working memory load effect (i.e., increase in CRT RT in the dual task compared to the single CRT task) and an interference effect (i.e., increase in misses in the dual task CRT vs. the single task CRT).

### Interpreting the DalCAB: Future assessments of validity

We have not formally examined the validity of our interpretation of the DalCAB here. Nonetheless, as the tasks included in the DalCAB are based on tasks purported to measure vigilance, orienting and executive control functions in cognitive psychology research, as a starting point, it is prudent to ensure that performance on each task is replicating performance patterns previously reported in the cognitive psychology literature. Overall, RT performance across the levels of the independent variables of interest in each DalCAB task followed the expected patterns, as summarized in Table 1. Therefore, our results might indirectly provide a preliminary assessment of the convergent validity of our interpretation of the DalCAB and, at the very least, justify a formal gathering of validity evidence for our interpretation of the DalCAB in the future.

The current findings are consistent with the interpretation of other analyses performed on these same data that examined the factor structure of performance on the DalCAB (Jones et al., 2015). Specifically, we used exploratory and confirmatory factor analysis to examine the preliminary factor structure and reliable common variance in RT and accuracy performance related to the variables incorporated into the DalCAB tasks. The resulting extracted model had 9 factors. We reported that: (1) each of the nine-factors are related to one of the vigilance, orienting and executive control networks and; (2) multiple measures derived from the same DalCAB tasks are associated with more than one factor, highlighting the importance of each of the specific tasks and measures selected from the DalCAB in measuring the functions of attention (Jones et al., 2015).

## Limitations

The conclusions we have drawn about the reliability of DalCAB performance are based on a small sample of healthy adults (*n* = 96). Our sample size is comparable to other studies exploring test-retest and split-half reliability for other computerized neuropsychological batteries (e.g., MicroCog: Raymond et al., 2006, *n* = 40, test-retest; CPT-PENN: Kurtz et al., 2001, *n* = 80, alternate forms; IntegNeuro: Williams et al., 2005, *n* = 21, test-retest (normative data base of over 1000); CogState: (Falleti et al., 2006), *n* = 45, test-retest and practice effects at a short time interval; *n* = 55, test-retest and practice effects at a longer time interval; Darby et al., 2002, *n* = 20 patients with mild cognitive impairment, *n* = 40 healthy control participants, test-retest; Collie et al., 2003, *n* = 113, test-retest). However, our sample is also homogeneous in nature, such that participants were recruited from a post-secondary environment, are highly educated, healthy and represent a small age range. As such, further research is needed before the normative data presented here can be interpreted in the context of other groups or in clinical settings. Ongoing research in our lab will gather evidence for the validation of our interpretation of the DalCAB (criterion and discriminant validity) by comparing the DalCAB to gold-standard neuropsychological tests of attention and examining the reliability of the DalCAB in healthy older adults (Rubinfeld et al., 2014). We have also recently submitted work on ensuring chronometric (clock) precision in the measurement of reaction time using computer-based tasks, using the DalCAB as an example (Salmon et al., submitted). Thus, the DalCAB holds promise for future use in research and clinical environments.

## Conclusions

Here, we sought to evaluate the test-retest reliability of the DalCAB over a short interval of 24-h. Our evaluation of the reliability and practice effects on performance of the tasks in the DalCAB indicate *Good* to *Very Good* test-retest reliability for mean RTs on all tasks and significant practice effects on mean RTs at 24-h after baseline for most tasks (5/7 tasks). We have also presented preliminary normative data that supports our interpretation of performance on the DalCAB, taking into account our small, homogenous sample. In particular, we report task-related effects that are consistent with those previously reported in the literature for similar tasks. Although validity was not formally assessed here, these preliminary findings might serve as pilot data, supporting future assessments of the convergent validity of our interpretation of the DalCAB as a measure of attention.

## Author contributions

SJ contributed to the conception of the study, established procedures for data analysis, aided in the analysis and interpretation of the data and aided in the preparation of the manuscript. BB contributed to the conception of the study, participated in the design of the study, aided in the analysis and interpretation of the data and aided in the preparation of the manuscript. FK participated in the acquisition of the data and aided in the preparation of the manuscript. AJ contributed to analysis of the data and aided in preparation of the manuscript. RK contributed to the conception of the study, the interpretation of the data and aided in preparation of the manuscript. GE contributed to the conception of the study, the interpretation of data, participated in the design of the study, and aided in the preparation of the manuscript. All authors read and approved the final manuscript.

## Funding

Funding for this research was provided by the Atlantic Canada Opportunities Agency, Atlantic Innovation Fund (to GE, RK), Springboard Technology Development Program Award (to GE), and the Dalhousie Department of Psychiatry Research Fund (to BB, GE).

### Conflict of interest statement

The authors declare that the research was conducted in the absence of any commercial or financial relationships that could be construed as a potential conflict of interest.

## Footnotes

^1^All tasks contained within the DalCAB employ a variation of playing cards or card suit stimuli, permitting direct comparison of performance across increasingly complex tasks. See (Figure 1).

^2^Due to methodological changes that resulted in a small sample size (*n* = 15), analysis of the location working memory task is not included in the current paper.

^3^We have indirectly compared the pattern of results/performance on the DalCAB to those reported by others using similar tasks purported to measure the components of attention as an initial step in exploring the validity of our interpretation of the DalCAB. Formal evidence of the validity of our interpretation of the DalCAB is needed before conclusions about our results can be generalized.

^4^The average skewness value across the analyzed DalCAB tasks presented here was 2.24 (range: 1.53–3.15). A previous analysis of these data in which more conservative lower and upper bound RT cutoffs were employed as a data cleaning method was carried out. Lower bound RT cutoff values were defined as the minimum reaction time at which 75% accuracy was achieved on each individual task, and ranged from 100 to 400 ms depending on task complexity (see Christie et al., 2013). Below these lower bound cutoffs, responses were considered anticipations and were excluded from analysis. Upper bound RT outliers on each task were defined within each participant using a *z*- score cut-off value of 3.29 (Tabachnick et al., 2001, pp. 67). Analysis of these cleaned data resulted in no change to the pattern of results or the assessments of test-retest reliability reported here. We also analyzed the data after applying an inverse transformation (i.e., 1/RT). Analysis of these transformed data was consistent with the analyses reported here with the following three exceptions: (1) a significant main effect of session in the SRT task (consistent with our already reported practice effect); (2) A significant interaction between gender and RSI in the SRT task; post hoc analyses revealed the same pattern of RT across RSI for both males and females, consistent with the pattern of RT across RSI reported above and; (3) longer RTs for females than males in the feature search task. No changes in the assessment of practice effects or test-retest reliability were found using the transformed data.

^5^The conclusions drawn here are based on a relatively homogenous sample of adults; further research is needed before the normative data presented here can be interpreted in the context of other groups or in clinical settings.
